# Efficacy and safety of Shatavari root extract (*Asparagus racemosus*) for menopausal symptoms: a randomized, double-blind, three-arm, placebo-controlled study

**DOI:** 10.3389/frph.2025.1654503

**Published:** 2025-11-27

**Authors:** John Ademola, Ashutosh Ajgaonkar, Tanisha Debnath, Khokan Debnath, Jayshree Langade

**Affiliations:** 1 San Francisco Research Institute, San Francisco, CA, United States; 2 Gynaecologic Division, Trupti Hospital, Thane West, India; 3 Obstetrics and Gynaecology, Davao Medical School Foundation, Davao, Philippines; 4 Medicine, Prakruti Hospital, Mumbai, India; 5 Deepak Dental Clinic, Thane, India

**Keywords:** menopause, *Asparagus racemosus*, *Withania somnifera*, perceived stress scale, menopause rating scale, menopause-specific quality of life questionnaire

## Abstract

Menopause brings hormonal changes that can significantly affect quality of life. Herbal remedies like Shatavari (*Asparagus racemosus* Willd.) and Ashwagandha (*Withania somnifera* L. Dunal) are Ayurvedic herbs traditionally used for hormonal balance and associated stress adaptation. The study evaluated the efficacy and safety of Shatavari root extract (SHT) alone and, in combination with Ashwagandha root extract (ARE-SHT), in women with menopausal symptoms. This was an 8-week, randomized, double-blind, placebo-controlled, multicentric study that enrolled 135 women aged 45–65 years. Participants were randomized into three groups: SHT (*n* = 45) or an ARE-SHT (*n* = 45), or a placebo (PL; *n* = 45). The primary outcome was evaluated by the change in the Menopause Rating Scale (MRS) score at weeks 4 and 8. Secondary outcomes were evaluated by changes in Perceived Stress Scale (PSS) score, Menopause-Specific Quality of Life Questionnaire (MENQOL) score, Profile of Mood States (POMS) score, hot flashes, and mood improvement. The serum hormonal assessments and safety evaluations (liver, renal, and thyroid function) were conducted at week 8. Baseline values were comparable among the groups. Significant improvements in MRS scores were observed in the ARE-SHT group compared to SHT [Week 4 and 8 (*p* < 0.0001)] and PL [weeks 4 (*p* = 0.01) and 8 (*p* < 0.0001)], respectively. MENQOL scores improved numerically by week 8. Esteem-related affect (*p* = 0.025) and mood improvement (*p* = 0.008) showed significant changes between the groups. Normal hormonal levels were maintained. Mild adverse events were reported by four participants (loose stools and dizziness in the ARE-SHT group, nausea in the SHT monotherapy group, and headache in the PL group). Shatavari root extract was safe and effective; however, combined with Ashwagandha root extract demonstrated a promising option in managing menopausal symptoms and in enhancing overall health. Future studies with longer duration and larger sample sizes across defined menopausal stages are required to confirm and extend these findings.

**Clinical Trial Registration:**

Clinicaltrials.gov, identifier [NCT06716554].

## 1 Introduction

Menopause is a natural biological phenomenon in women, typically occurring between the ages of 45 and 55 years (1). It is characterised by the persistent cessation of menstruation for at least 12 consecutive months, followed by a decline in ovarian follicles (2), which leads to decreased levels of estradiol, along with increased follicle-stimulating hormone (FSH), ultimately resulting in estrogen deficiency. Menopause is a natural physiological process, frequently accompanied by symptoms that can significantly impact a woman's quality of life (3). Previous studies have reported that approximately 60% of women with menopause experience mild symptoms, 20% of them report severe symptoms, and 20% remain asymptomatic. The common menopausal symptoms are hot flashes, excessive sweating, reduced libido, sleep disturbances, urogenital issues (vaginal dryness, painful intercourse, and recurrent bladder infections), breast tenderness, joint pain, cognitive changes, and mood disturbances, including depression and anxiety symptoms (4). The available treatments for these symptoms are hormone therapy (HT), prescription antidepressants, physical activity, and lifestyle modifications (5).

Ayurveda, the traditional system of medicine in India, has long utilized adaptogenic herbs to support female reproductive health and hormonal balance. Among these, Shatavari (*Asparagus racemosus* Willd.) and Ashwagandha (*Withania somnifera* L. Dunal) are the two most extensively used botanical therapies for hormone regulation and stress adaptation. Shatavari is a well-known herb in Ayurveda for its phytoestrogenic and rejuvenating properties. It is commonly known as Wild Asparagus or Indian Asparagus. It is referred to as the “Queen of Herbs”. It was traditionally said to foster attachment and affection. Shatavari is believed to exert its effects through phytoestrogenic activity of its steroidal saponins (Shatavarins), which have affinity for estrogen receptors and may modulate gonadotropin levels, in addition to antioxidant and anti-inflammatory actions (6). Shatavari acts as a primary rejuvenative herb for women, helps to regulate hormonal balance, improve reproductive health, and reduce menopausal symptoms such as hot flashes, insomnia, and mood disturbances. Additionally, it supports adrenal function and helps to maintain healthy estrogen levels during menopause to relieve menopausal symptoms such as hot flashes, insomnia, depression, and excessive sweating (7). Shatavari root extract contains various phytoconstituents, including Shatavarins, which are steroidal saponins in structure and have affinity towards estrogen receptors (8) and exhibit estrogen-like activity, which helps in regulating overall hormonal regulation. It offers more benefits, such as supporting heart health, a healthy mood, aiding digestion, and having antioxidant and anti-inflammatory effects (6).

Ashwagandha, another well-known, revered herb in Ayurveda, accompanies Shatavari with its adaptogenic properties. Ashwagandha acts primarily through modulation of the hypothalamic-pituitary-adrenal (HPA) axis, reducing cortisol levels and enhancing stress resilience, alongside potential neuromodulatory and reproductive health benefits. It includes withanolides, sitoindosides, flavonoids, and steroidal lactones (9). Ashwagandha has been extensively studied for its effects, such as helping the body adapt to stress, and its role in hormonal regulation (10). In healthy women, it is still an area that needs further research. When used in combination with Shatavari, the two herbs may offer additive benefits, particularly in promoting women's sexual well-being (11, 12). Ashwagandha also supports reproductive function, aids in promoting a healthy balance in sexual and reproductive health (13). Although both Shatavari and Ashwagandha have been traditionally used for supporting women's health, the scientific evidence on their role in managing menopausal symptoms remains limited, particularly regarding their combined use at the extract concentrations of Shatavarins (>10%) and Withanolides (>5%) (7, 10, 14).

This prospective, randomized, double-blind, three-arm, placebo-controlled study assessed and compared the efficacy and safety of Shatavari alone and Shatavari combined with Ashwagandha, in comparison to placebo.

## 2 Materials and methods

### 2.1 Study design

**Figure 1:**
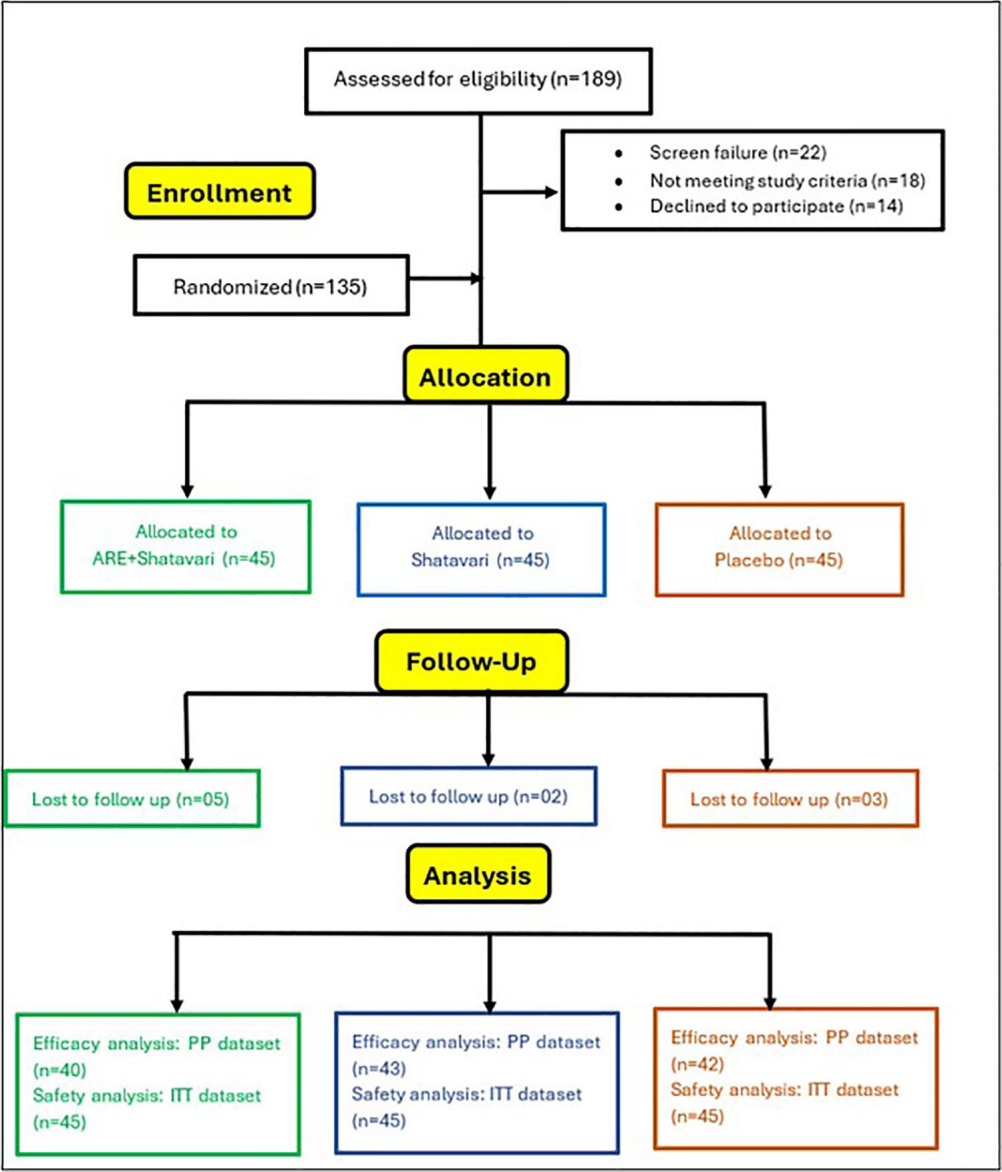
CONSORT flow-diagram. Per-protocol analyses included Menopause Rating Scale, Perceived Stress Scale, Menopause-Specific Quality of Life, Profile of Mood States, hot flashes, and mood improvement, whereas serum hormone and laboratory safety parameters (liver, renal, and thyroid) were evaluated for the intention-to-treat population.

This was an eight-week, prospective, multicenter, randomized, three-arm, double-blind, placebo-controlled, parallel clinical trial designed to compare the safety and efficacy of the Shatavari root extract alone in comparison to a combination of Ashwagandha root extract and Shatavari root extract, and placebo, for treating menopausal symptoms in women (Figure 1). The combination arm was selected to evaluate the additive effects. The trial was conducted at Dr. D.Y. Patil Medical College & Hospital, Navi Mumbai, India and San Francisco Research Institute, California. The study was conducted between October 4, 2024 and December 22, 2024. Written informed consent was obtained from all participants in their preferred language before enrollment. The enrollment of participants commenced on October 4, 2024. A comprehensive explanation of the study's objectives and expected outcomes was provided to each participant before obtaining consent.

### 2.2 Ethical approvals

The clinical study protocol was approved by the Institutional Ethics Committees (IEC) of Dr. D.Y. Patil Medical College & Hospital, Navi Mumbai, Maharashtra, India (IEC Reference No: DYP/IECBH/2024/424) and the ALLENDALE investigational review board (Protocol number: SHT-1076-2024-01; approval date: 1 October 2024). The trial was prospectively registered with the Clinical Trials Registry of India (CTRI) with registration number CTRI/2024/10/074570 dated October 1, 2024 (https://ctri.nic.in/Clinicaltrials/pmaindet2.php?EncHid=MTE0ODE1&Enc=&userName=), and with clinicaltrials.gov with registration number NCT06716554 (https://clinicaltrials.gov/study/NCT06716554) dated December 4, 2024. The study was conducted as per the ethical principles outlined in the Declaration of Helsinki (2013 revision), Good Clinical Practice (GCP) guidelines, and the Consolidated Standards of Reporting Trials (CONSORT) statement.

### 2.3 Study population and eligibility criteria

#### 2.3.1 Inclusion criteria

Women aged 45–65 years with an intact uterus and ovaries, who had experienced irregular menstrual cycles in the past 12 months were included. Irregularities were defined as cycles delayed or advanced by more than seven days, missing at least two cycles in the past year, or absence of menstruation for at least 60 days. Participants who had reported menopausal symptoms, such as hot flashes, insomnia, migraines, and irritability, with a body mass index (BMI) between 18 and 35 kg/m^2^ were included in the study.

#### 2.3.2 Exclusion criteria

Prior use of herbal extracts or HT for more than three months before the study. Participants were also excluded if they had active medical, surgical, or gynecological conditions; a history of alcohol, tobacco, or substance abuse; or clinically significant diseases affecting the cardiovascular, gastrointestinal, hepatic, neurological, endocrine, hematologic, or other systemic systems that could affect the study protocol or its outcomes. Women with a history of bilateral ovariectomy or breast or cervical cancer were not eligible. Additional exclusion criteria were mental health conditions that could impair the participant's ability to comprehend the study, uncooperative behavior, or an inability to attend follow-up visits. Uncontrolled infections or other medical issues that might interfere with the study. Furthermore, participants with known hypersensitivity to Ashwagandha or Shatavari, those who had participated in another clinical trial within the last three months, or those with any condition that could compromise their safe participation in the study were excluded.

Participants could be withdrawn from the study under specific circumstances, including voluntary withdrawal of consent, occurrence of adverse events deemed significant by the clinical investigator, or the discovery of any unexpected, significant, or unacceptable risk to enrolled subjects. Withdrawal was also warranted in cases of significant non-compliance with study procedures, protocol violations, or any other reason considered likely to affect study outcomes or participant safety. In addition, participants were withdrawn if the study itself was terminated.

After providing informed consent, 135 women who met the inclusion criteria were enrolled in the study.

### 2.4 Sample size calculation

The calculation was based on the expected reduction in Menopause Rating Scale (MRS) total scores (0–44 scale) with Ashwagandha 600 mg/day as reported in prior studies. Power calculations were performed using data from a previous study by Gopal et al. (12), which reported a reduction in MRS scores with Ashwagandha 600 mg/day was −3.37 (95% C.I.: −4.10 to −2.73). It was assumed that the improvement in MRS scores with Shatavari root extract and combination therapy (Ashwagandha plus Shatavari root extracts) would be similar to Ashwagandha monotherapy in Gopal et al. (12) study. A sample size of 39 resulted in a statistical test power of 90.7%. Based on these assumptions, a sample size of a total of 39 (13 in each arm) completed cases was needed to assess the study objective at 80% power and 5% level of significance with 1:1:1 allocation. To account for a potential attrition rate of approximately 30% and to increase the precision of the treatment effect estimates, the planned recruitment target was set at 90 participants (30 per arm). Ultimately, 135 participants were enrolled, which further strengthened the robustness of the study findings, minimized the impact of attrition, and allowed exploratory subgroup and covariate-adjusted analyses. Per-protocol (PP) analyses included MRS, Perceived Stress Scale (PSS-10), Menopause-Specific Quality of Life (MENQOL), Profile of Mood States (POMS), hot flashes, and mood improvement, whereas serum hormone and laboratory safety parameters (liver, renal, thyroid) were evaluated for the intention-to-treat (ITT) population.

#### 2.4.1 Randomization and blinding

Block randomization was carried out using an automated random number generation system (Rando version 1.2 R), pre-specified for the study. Participants underwent assessments at baseline, week 4, and week 8. To ensure blinding, the Shatavari, Shatavari combination (Ashwagandha and Shatavari), and placebo capsules were identical in appearance, shape, color, and packaging. The randomization codes were securely stored in separate envelopes and were only accessed by the investigator after assigning a study number to each participant. The investigator and all personnel involved in data collection and statistical analysis remained blinded to the treatment allocation until all study results were entered into the locked raw data file, after which the blinding code was released.

### 2.5 Study interventions

Dried roots of *Asparagus racemosus* Willd. (Asparagaceae; Asparagi racemosi radix) and *Withania somnifera* (L.) Dunal (Solanaceae; Withaniae radix) were used in this study. Participants were randomized in a 1:1:1 ratio to receive either a 300 mg Shatavari root extract capsule (SHT-300 mg) (Ixoreal Biomed Inc., Los Angeles, USA), a combination capsule containing 300 mg Shatavari and 250 mg Ashwagandha (SHT-300 mg-ARE-250 mg) root extracts, or an identical placebo capsule (PL-300 mg). Participants were instructed to take one capsule daily after breakfast with water for 8 weeks.

### 2.6 Investigational products

Both Shatavari and Ashwagandha root extracts are commercially available products. The extracts were obtained in alignment with the green chemistry principles, devoid of any harsh solvents, in a current good manufacturing practice (cGMP)-certified facility. The Shatavari extract is standardized to a total Shatavarin content of >10% by high-performance liquid chromatography (HPLC) (Supplementary Figure S1) with an herb to extract ratio of 13:1. The Shatavari root extract has a yellowish-brown powder-like appearance. Ashwagandha root extract contains Withanolides concentration of >5% standardized by HPLC (Supplementary Figure S2) with an herb to extract ratio of 12:1. The placebo group received starch, and the placebo capsules were of the same size, shape, odor, color, and taste as the Shatavari capsules. The Shatavari root extract used in this study has been classified as Extract Type A in accordance with the Consensus statement on the reporting of pharmacology and physiology studies in natural product research [ConPhyMP; (15)]. The classification was confirmed using the ConPhyMP interactive tool (https://ga-online.org/best-practice/#conphymp) under the domain of Phytochemical Characterization of Medicinal Plant Extracts.

### 2.7 Outcome measures

Participants were assessed at baseline, week 4, and week 8 by a trained clinician. At each visit, vital signs were recorded, including systolic and diastolic blood pressure, pulse rate, respiratory rate, and body temperature (16).

#### 2.7.1 Primary outcome measure

The MRS is an 11-item questionnaire, ranging from no symptoms to very severe symptoms. Total scores for the MRS range from 0 (asymptomatic) to 44 (highest degree of complaints). The three domains include: psychological, somato-vegetative, and urogenital, for which the minimal/maximal scores vary depending upon the symptoms (13, 17).

#### 2.7.2 Secondary outcome measures

##### 2.7.2.1 Perceived stress scale (PSS-10)

The PSS-10 is a widely used psychological instrument for assessing the perception of stress. It evaluates the extent to which situations in life are perceived as unpredictable, uncontrollable, and overwhelming. The scale consists of items designed to measure how individuals appraise their daily experience as stressful. Based on their scores, participants are classified into three groups: low stress, moderate stress, and high stress, providing a structured assessment of stress perception (18, 19).

##### 2.7.2.2 Menopause-specific quality of life questionnaire (MENQOL)

The MENQOL, developed in 1996, is an administered tool designed to assess health-related quality of life in the immediate post-menopausal period. It consists of 29 items presented in a Likert-scale format, each evaluating the impact of menopausal symptoms over the past month. The questionnaire covers four key domains: vasomotor, psychosocial, physical, and sexual, providing a comprehensive assessment of how menopause affects daily life and well-being.

##### 2.7.2.3 Profile of mood states (POMS)

The POMS is a 40-item questionnaire rated on a 5-point Likert Scale, designed to assess an individual's current emotional state. It measures scores across several mood dimensions, including Tension, Anger, Fatigue, Depression, Esteem-related affect (ERA), Vigor, and Confusion. The Total Mood Disturbance (TMD) is calculated by summing the totals for the negative subscales (tension, confusion, anger, fatigue, depression) and subtracting the totals of the positive subscales (ERA, vigour), providing an overall measure of mood disturbance.

##### 2.7.2.4 Hot flashes and mood improvement

The assessment of hot flashes and mood improvement was conducted using specific items from the MRS. Hot flashes were evaluated based on their frequency and intensity, while mood improvement was assessed by measuring changes in emotions such as irritation, anxiety, and despair. Participants rated the severity of each symptom on a scale from 0 (not bothersome) to 4 (very troublesome) over the past month. Changes in hot flashes and mood were analyzed by comparing symptom ratings recorded at the beginning and end of the study.

##### 2.7.2.5 Hormonal and laboratory outcomes

Blood samples were collected at baseline and week 8 to assess serum hormonal outcomes [serum estradiol, Follicle-Stimulating Hormone [FSH], luteinizing hormone (LH), and testosterone]. Additionally, liver function parameters (serum alanine transaminase [ALT], aspartate transaminase [AST], alkaline phosphatase [ALP], bilirubin), renal function markers (serum creatinine, blood urea nitrogen), and thyroid function parameters (triiodothyronine [T3], thyroxine [T4], and thyroid-stimulating hormone [TSH]) were measured. Samples were drawn into both ethylene diamine tetra acetic acid (EDTA) and non-EDTA vials, centrifuged, and the serum was stored at −80°C for later analysis. Hormonal concentrations were determined using enzyme-linked immunosorbent assay (ELISA) kits.

##### 2.7.2.6 Safety assessment

Treatment-Emergent Adverse Events (TEAEs) and Treatment-Emergent Serious Adverse Events (TESAEs), whether observed by the physician or reported by the participant, were recorded for all the participants at week 4 and week 8. Additionally, changes in liver, thyroid, and renal parameters were evaluated by comparing baseline values to those recorded in week 8.

### 2.8 Statistical analysis

Data were analyzed using the Stata 13.1, statistical software for Windows. Descriptive statistics were calculated for continuous variables, including means and standard deviations (SD). A 95% confidence interval (CI) was used to ensure adherence to the best statistical techniques. All analyses were conducted using two-sided tests with statistical significance set at a *p*-value of less than 0.05. Between-group comparisons were analysed using a one-way analysis of variance (ANOVA) with *post hoc* Tukey's test for pairwise comparisons. Categorical variables were analyzed using the Chi-square test across the groups (SHT vs. ARE-SHT vs. Placebo), and results were presented as the number and percentage of participants in each category, including a category for missing data if necessary. Due to baseline differences in key covariates (age, menopausal status, BMI, and FSH), a generalized linear model (GLM) was applied. Intervention type (study group) was specified as the main between-subjects factor, and baseline covariates were included in the model to adjust for potential confounding. This approach provided adjusted estimates of treatment effects while accounting for variability in baseline characteristics.

## 3 Results

A total of 135 participants were randomized to receive SHT (*n* = 45), ARE-SHT (*n* = 45), or PL (*n* = 45). During the study period, ten participants were lost to follow-up (Figure 1). As a result, 125 participants were included in the efficacy analysis (PP dataset), while 135 participants were analyzed for both safety and efficacy (ITT dataset). The primary reasons included withdrawal of consent due to personal reasons (*n* = 4), non-compliance with study visits or procedures (*n* = 3), and loss of contact despite repeated attempts (*n* = 3). No participant discontinued due to adverse events. The CONSORT flow chart is depicted in Figure 1.

### 3.1 Profile of all parameters for participants enrolled in the ITT dataset (*n* = 135)

The study findings showed no significant differences among the three groups (SHT, ARE-SHT, and PL) in most parameters (Table 1). The mean age was 50.84 ± 4.17 years (ARE-SHT), 52.13 ± 5.36 years (SHT), and 50.87 ± 3.97 years (PL; *p* = 0.308). BMI was comparable across groups (*p* = 0.878). No significant differences were observed in systolic blood pressure (*p* = 0.786), diastolic blood pressure (*p* = 0.705), pulse rate (*p* = 0.603), body temperature (*p* = 0.365), or respiratory rate (*p* = 0.239). The MRS total score (*p* = 0.896) and PSS-10 (*p* = 0.437) were similar among groups. The distribution of menopause status (perimenopausal vs. postmenopausal) was comparable across the groups, with no significant differences observed (*p* = 0.918).

**Table 1:** Demography and baseline profile of participants in ITT dataset (*n* = 135).

Parameters	ARE-SHT (*n* = 45)	SHT (*n* = 45)	PL (*n* = 45)	ANOVA
	Mean (SD.)	Mean (SD.)	Mean (SD.)	“*p*”*
Demography				
Age (years)	50.84 (4.17)	52.13 (5.36)	50.87 (3.97)	0.308
BMI (kg/m^2^)	25.91 (3.55)	25.90 (4.02)	25.53 (4.56)	0.878
Physical examination				
SBP (mmHg)	114.33 (5.56)	113.80 (4.78)	114.4 (74.00)	0.786
DBP (mmHg)	74.89 (4.34)	75.38 (4.53)	75.64 (4.16)	0.705
Pulse rate (per min)	76.49 (7.55)	76.16 (6.63)	77.67 (8.17)	0.603
Body temperature (°F)	98.12 (0.69)	97.98 (0.76)	97.91 (0.72)	0.365
Respiratory rate (per min.)	17.44 (2.17)	16.69 (2.21)	17.20 (2.06)	0.239
Menopause Status				
Perimenopause	5 (3.70%)	4 (2.96%)	4 (2.96%)	0.918
Post menopause	40 (29.63%)	41 (30.37%)	41 (30.37%)	
MRS				
• Somato-vegetative domain	10.04 (3.91)	10.51 (3.32)	10.58 (3.44)	0.742
• Psychological domain	10.89 (4.00)	10.56 (3.13)	10.73 (3.39)	0.904
• Urogenital domain	7.42 (3.47)	8.00 (3.00)	7.91 (2.88)	0.642
• MRS total Score	28.36 (10.55)	29.07 (8.45)	29.22 (8.94)	0.896
PSS-10				
• PSS total score	23.33 (6.92)	24.31 (3.76)	24.69 (4.16)	0.437
MENQOL				
• Vasomotor	3.43 (1.36)	3.61 (1.10)	3.50 (1.32)	0.799
• Psychosocial	3.07 (1.19)	3.32 (0.99)	3.06 (1.08)	0.443
• Physical	2.76 (1.12)	3.23 (0.92)	3.14 (1.00)	0.072
• Sexual	2.91 (1.63)	2.70 (1.56)	2.98 (1.61)	0.684
• MENQOL total score	2.92 (1.05)	3.24 (0.83)	3.14 (0.96)	0.274
POMS				
• Tension	6.58 (4.90)	6.33 (5.31)	6.22 (4.76)	0.942
• Anger	7.18 (4.30)	5.67 (3.12)	7.11 (4.33)	0.127
• Depression	7.20 (5.38)	6.11 (4.97)	5.67 (4.58)	0.328
• Fatigue	8.58 (3.92)	9.22 (3.23)	9.27 (3.13)	0.571
• Confusion	5.87 (4.09)	7.56 (3.73)	5.78 (3.15)	0.038
• Esteem-related affect	12.44 (3.94)	12.00 (3.59)	12.31 (3.93)	0.852
• Vigour	8.09 (2.95)	7.49 (2.78)	8.76 (3.27)	0.140
• Total mood disturbance	114.87 (19.16)	115.40 (20.32)	112.98 (18.36)	0.822
Recording of Hot flashes improvement				
• Hot flashes	15.78 (3.75)	15.58 (3.09)	16.11 (4.88)	0.813
Recording of Mood improvement				
• Mood improvement	14.56 (3.78)	14.51 (3.89)	15.84 (5.03)	0.247

ARE, ashwagandha root extract; BMI, body mass index; DBP, diastolic blood pressure; ITT, intent-to-treat; SHT, shatavari; PL, placebo; SBP, systolic blood pressure; PSS, perceived stress scale; POMS, profile of mood states; MRS, menopause rating scale; MenQol, menopause-specific quality of life; SD., standard deviation; Yrs, years; Kg, kilogram; m, meters; mmHg, millimeters of mercury; min, minutes; °F, degree Fahrenheit.

* One-way analysis of variance (ANOVA) pairwise comparisons.

**Table 2:** MRS scale and MEN-QoL scale at baseline and during the study period in the PP dataset (*n* = 125).

Parameters	ARE-SHT (*n* = 40)	SHT (*n* = 43)	PL (*n* = 42)	ANOVA	ARE-SHT vs. SHT			SHT vs. PL			ARE-SHT vs. PL		
	Mean (SD.)	Mean (SD.)	Mean (SD.)	“*p*”**	Mean Diff.	Effect size (d)	“*p*”*	Mean Diff.	Effect size (d)	“*p*”*	Mean Diff.	Effect size (d)	“*p*”*
BMI (kg/m^2^)													
• Baseline	26.09 (3.58)	25.58 (3.71)	25.36 (4.54)	0.698	–	–	1.000	–	–	1.000	–	–	1.000
• Change at week 4	−0.40 (1.00)	0.26 (1.16)	0.63 (1.30)	<0.0001	−0.67	−0.671	0.030	−0.37	−0.317	0.444	−1.04	−1.039	<0.0001
• Change at week 8	−1.63 (2.77)	−0.37 (3.25)	0.17 (1.28)	0.006	−1.26	−0.456	0.083	−0.54	−0.167	1.000	−1.81	−0.652	0.006
MRS scale													
Somato-vegetative domain													
• Baseline	9.98 (3.72)	10.67 (3.24)	10.50 (3.49)	0.640	–	–	1.000	–	–	1.000	–	–	1.000
• Change at week 4	−2.13 (2.69)	−2.21 (2.58)	−1.14 (2.80)	0.135	0.08	0.031	1.000	−1.07	−0.414	0.695	−0.98	−0.365	0.145
• Change at week 8	−5.03 (3.07)	−3.86 (2.94)	−1.50 (3.56)	<0.0001	−1.16	−0.380	0.047	−2.36	−0.803	0.013	−3.53	−1.149	<0.0001
Psychological domain													
• Baseline	10.98 (3.83)	10.58 (3.13)	10.69 (3.33)	0.866	–	–	1.000	–	–	1.000	–	–	1.000
• Change at week 4	−3.18 (2.47)	−2.33 (2.89)	−0.45 (3.13)	<0.0001	−0.85	−0.344	1.000	−1.87	−0.648	0.038	−2.72	−1.103	0.008
• Change at week 8	−5.10 (3.09)	−3.93 (3.59)	−1.07 (3.30)	<0.0001	−1.17	−0.379	1.000	−2.86	−0.795	0.001	−4.03	−1.305	<0.0001
Urogenital domain													
• Baseline	7.63 (3.18)	7.95 (3.05)	7.81 (2.88)	0.886	–	–	1.000	–	–	1.000	–	–	1.000
• Change at week 4	−2.73 (2.22)	−1.91 (2.20)	−0.43 (2.74)	<0.0001	−0.82	−0.369	0.360	−1.48	−0.671	0.202	−2.30	−1.035	0.003
• Change at week 8	−2.95 (2.28)	−2.37 (2.21)	−0.69 (2.47)	<0.0001	−0.58	−0.254	0.598	−1.68	−0.759	0.086	−2.26	−0.993	0.002
MRS total Score													
• Baseline	28.58 (9.86)	29.21 (8.49)	29.00 (8.89)	0.949	–	–	1.000	–	–	1.000	–	–	1.000
• Change at week 4	−8.03 (5.80)	−6.44 (5.09)	−2.02 (7.41)	<0.0001	−1.58	−0.273	0.902	−4.42	−0.868	0.144	−6.00	−1.034	0.010
• Change at week 8	−13.08 (6.56)	−13.07 (6.87)	−3.26 (7.97)	<0.0001	−0.01	−0.001	1.000	−9.81	−1.427	<0.0001	−9.81	−1.495	<0.0001
MEN-QoL scale													
Vasomotor domain													
• Baseline	3.49 (1.27)	3.57 (1.09)	3.49 (1.27)	0.938	–	–	1.000	–	–	1.000	–	–	1.000
• Change at week 4	−0.55 (1.60)	−0.73 (1.37)	−0.57 (1.52)	0.836	0.18	0.112	1.000	−0.16	−0.115	1.000	0.02	0.013	1.000
• Change at week 8	−1.30 (1.22)	−1.05 (1.66)	−0.82 (1.90)	0.408	−0.25	−0.207	0.941	−0.23	−0.138	1.000	−0.48	−0.394	0.450
Psychosocial domain													
• Baseline	3.07 (1.24)	3.31 (0.99)	3.07 (1.10)	0.513	–	–	0.978	–	–	0.939	–	–	1.000
• Change at week 4	−0.30 (0.92)	−0.62 (1.24)	−0.32 (0.93)	0.299	0.32	0.345	1.000	−0.30	−0.240	1.000	0.02	0.021	1.000
• Change at week 8	−0.82 (0.95)	−0.66 (1.42)	−0.51 (1.31)	0.531	−0.16	−0.165	0.626	−0.15	−0.108	1.000	−0.31	−0.328	0.998
Physical domain													
• Baseline	2.85 (1.10)	3.23 (0.93)	3.12 (1.03)	0.216	–	–	0.264	–	–	1.000	–	–	0.682
• Change at week 4	−0.48 (0.59)	−0.60 (1.00)	−0.37 (0.80)	0.457	0.11	0.196	0.917	−0.22	−0.223	1.000	−0.11	−0.184	0.458
• Change at week 8	−0.93 (0.58)	−0.62 (0.99)	−0.51 (1.14)	0.113	−0.32	−0.553	0.027	−0.10	−0.104	1.000	−0.42	−0.732	0.031
Sexual domain													
• Baseline	2.95 (1.60)	2.73 (1.53)	2.93 (1.66)	0.782	–	–	1.000	–	–	1.000	–	–	1.000
• Change at week 4	−0.59 (1.08)	−0.29 (1.40)	−0.32 (1.56)	0.552	−0.30	−0.274	1.000	0.02	0.016	1.000	−0.27	−0.253	1.000
• Change at week 8	−0.90 (1.02)	−0.43 (1.22)	−0.48 (1.74)	0.238	−0.47	−0.455	1.000	0.04	0.034	1.000	−0.42	−0.414	0.760
MENQOL total score													
• Baseline	2.98 (1.06)	3.23 (0.84)	3.13 (0.99)	0.482	–	–	0.687	–	–	1.000	–	–	1.000
• Change at week 4	−0.46 (0.48)	−0.58 (0.87)	−0.38 (0.71)	0.397	0.13	0.265	1.000	−0.21	−0.240	1.000	−0.08	−0.168	1.000
• Change at week 8	−0.94 (0.55)	−0.65 (0.93)	−0.54 (1.10)	0.117	−0.29	−0.524	0.133	−0.11	−0.122	1.000	−0.40	−0.730	0.131

BMI, body mass index; PP, per protocol; ARE/SHT, ashwagandha root extract/shatavari; SHT, shatavari; SD., standard deviation; PL, placebo; MRS, menopause rating scale; Men-Qol, menopause-specific quality of life; Kg, kilogram; m, meters.

* One-way analysis of variance (ANOVA) with Bonferroni *post hoc* pairwise comparisons.

** Between group *p*-value (ANOVA).

MENQOL subdomains, including vasomotor (*p* = 0.799), psychosocial (*p* = 0.443), physical (*p* = 0.072), and sexual (*p* = 0.684), showed no significant differences. The only significant difference was observed in the POMS confusion subdomain (*p* = 0.038). Mood improvement (*p* = 0.247) and hot flash reduction (*p* = 0.813) were also not significantly different among groups. Table 1 presents the baseline findings.

### 3.2 Menopause rating scale and menopause-specific quality of life questionnaire

The individual change for the domains (psychological and urogenital) in week 4 was also highly significant (*p* < 0.0001). The total MRS score change in weeks 4 and 8 was highly significant (*p* < 0.0001) between groups. The difference observed was highly significant for comparisons of SHT vs. PL and ARE-SHT vs. PL (Table 2). Within each group, MENQOL total scores improved from baseline to week 8 (ARE-SHT: −0.94, SHT: −0.65, PL: −0.54); however, between-group comparisons did not demonstrate statistical significance (Table 2). Adjustment for baseline covariates (age, menopause status, BMI, and FSH) confirmed that the observed effects were independent of demographic factors, with FSH emerging as the only significant covariate across all MRS and MENQOL domains (*p* < 0.001; Supplementary Table S1).

**Figure 2:**
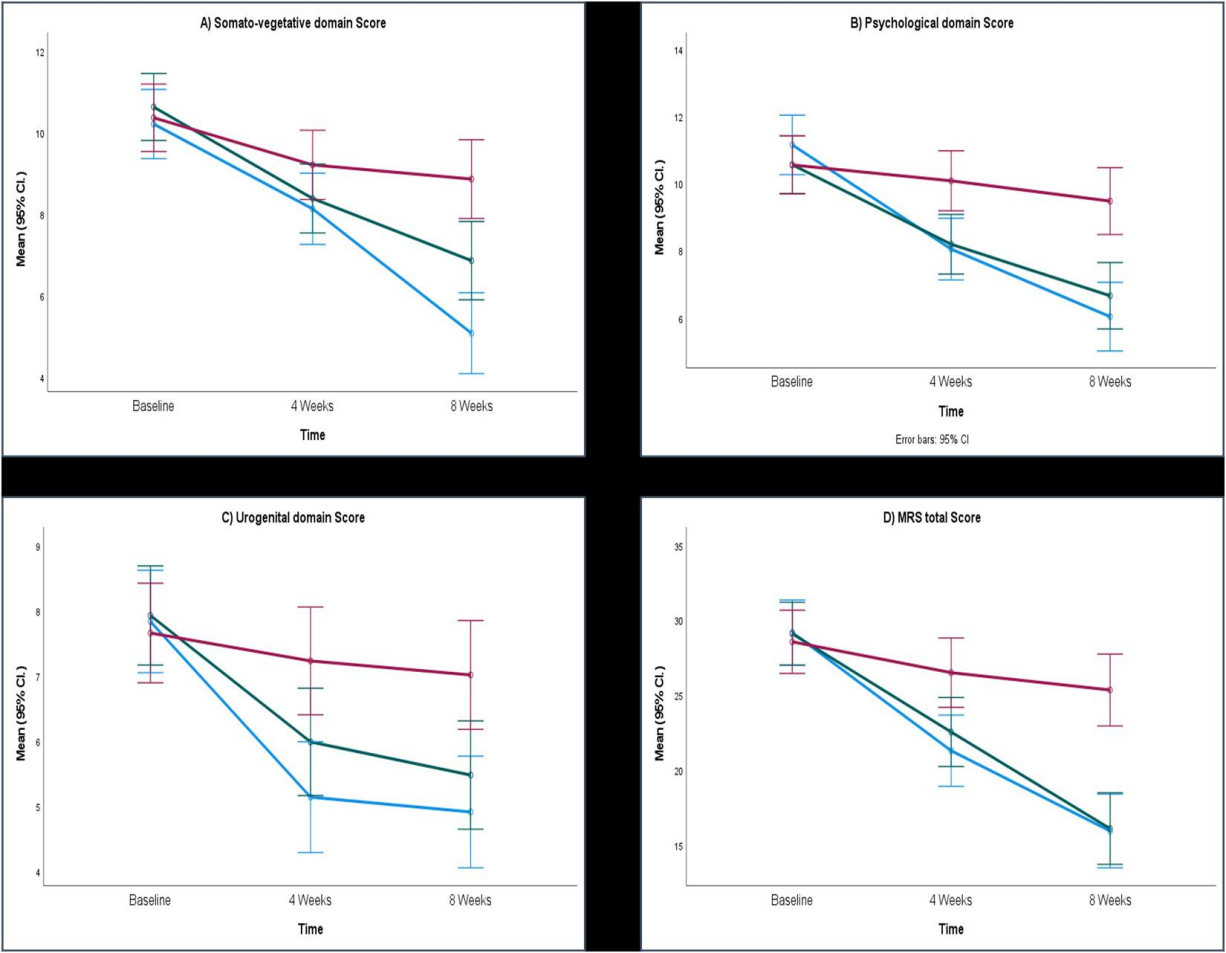
Mean scores of MRS. **(A)** Somato-vegetative domain score, **(B)** psychological domain score, **(C)** urogenital domain score and **(D)** total MRS score. ARE: Ashwagandha root extract; SHT, Shatavari; PL, placebo; MRS, menopause rating scale; CI, confidence interval.

**Figure 3:**
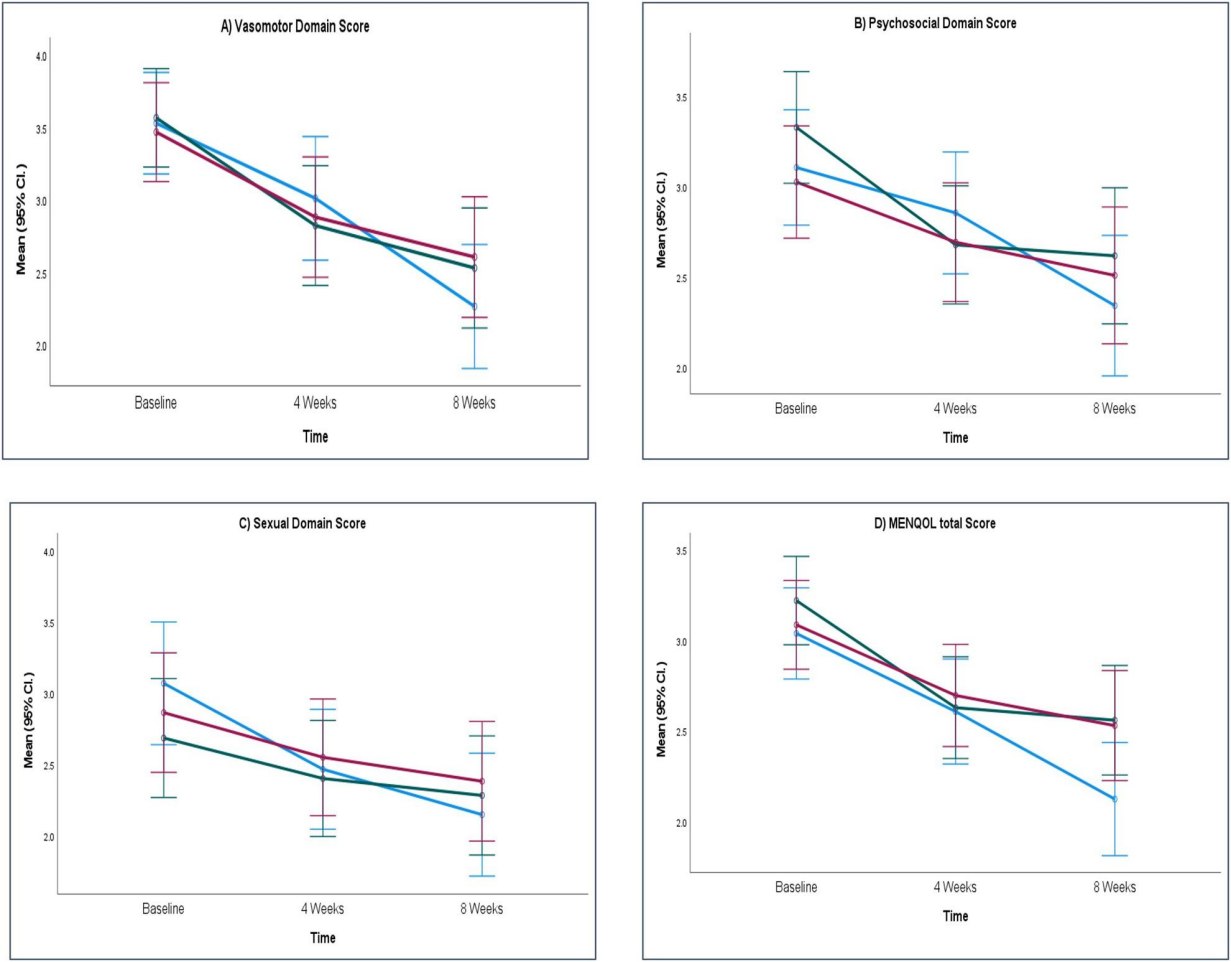
Mean scores of MENQOL assessment. **(A)** Vasomotor domain score, **(B)** psychological domain score, **(C)** sexual domain score and **(D)** MENQOL total score. ARE, ashwagandha root extract; SHT, shatavari; PL, placebo; MENQOL, Menopause-specific Quality of Life Questionnaire; CI, Confidence Interval.

The mean scores for MRS at week 4 and week 8 are depicted in Figure 2. Figure 3 shows the MENQOL assessment scores at week 4 and week 8.

### 3.3 Profile of mood states

**Figure 4:**
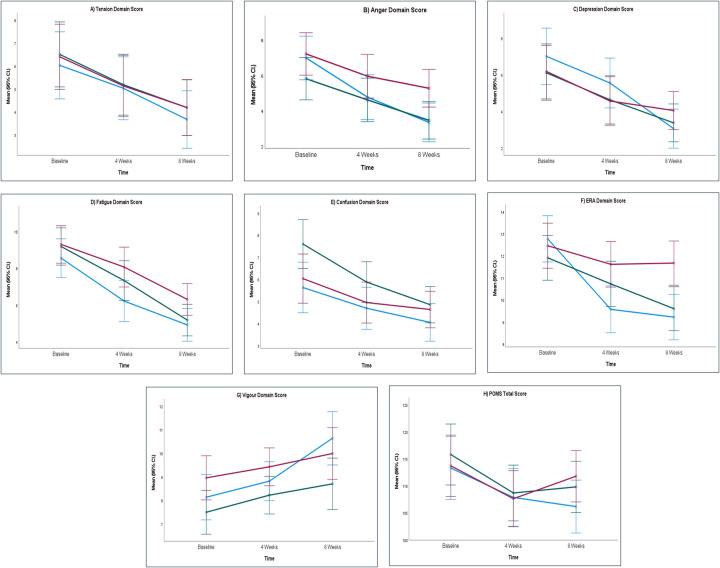
Mean scores of POMS assessment. **(A)** Tension domain score, **(B)** anger domain score, **(C)** depression domain score, **(D)** fatigue domain score, **(E)** confusion domain score, **(F)** ERA domain score, **(G)** vigour domain score and **(H)** POMS total score. ARE, ashwagandha root extract; SHT, shatavari; PL, placebo; POMS, profile of mood states; CI, confidence interval.

At week 4 ARE-SHT showed a numerical improvement [−6.20 (18.77)] compared to SHT [−6.51 (15.56)] and PL [−5.98 (16.43)]. Following week 8, ARE-SHT also demonstrated significant improvement [−6.80 (18.09)] relative to PL [−2.12 (19.67)]. No other domains or overall POMS scores showed significant changes across the groups (Table 3). As per the adjusted values, FSH emerged as a significant predictor for tension (*p* = 0.002), ERA (*p* < 0.001), and total POMS score (*p* < 0.0001), while age, BMI, and menopause status did not significantly influence outcomes. Adjusted mean scores along with 95% confidence intervals are presented in Supplementary Table S2 to indicate the precision of these estimates. The mean scores for POMS at week 4 and week 8 are depicted in Figure 4.

**Table 3:** POMS scale during the study period in the PP dataset (*n* = 125).

Parameters	ARE-SHT (*n* = 40)	SHT (*n* = 43)	PL (*n* = 42)	ANOVA	ARE-SHT vs. SHT			SHT vs. PL			ARE-SHT vs. PL		
	Mean (SD.)	Mean (SD.)	Mean (SD.)	“*p*”**	Mean Diff.	Effect size (d)	“*p*”*	Mean Diff.	Effect size (d)	“*p*”*	Mean Diff.	Effect size (d)	“*p*”*
POMS scale													
Tension domain													
• Baseline	6.33 (4.80)	6.40 (5.39)	6.21 (4.64)	0.986	‒	‒	1.000	‒	‒	1.000	‒	‒	1.000
• Change at week 4	−1.15 (3.95)	−1.28 (4.07)	−1.17 (3.89)	0.987	0.13	0.033	1.000	−0.11	−0.028	1.000	0.02	0.004	1.000
• Change at week 8	−2.48 (4.45)	−2.40 (4.92)	−2.02 (5.39)	0.906	−0.08	−0.018	1.000	−0.37	−0.076	1.000	−0.45	−0.101	1.000
Anger domain													
• Baseline	7.13 (4.18)	5.74 (3.17)	7.14 (4.37)	0.176	‒	‒	0.217	‒	‒	0.257	‒	‒	1.000
• Change at week 4	−2.40 (5.17)	−1.05 (3.37)	−1.24 (4.72)	0.336	−1.35	−0.262	0.510	0.19	0.057	1.000	−1.16	−0.225	0.723
• Change at week 8	−3.73 (4.26)	−2.35 (3.65)	−1.86 (5.46)	0.158	−1.38	−0.323	0.504	−0.49	−0.135	1.000	−1.87	−0.438	0.191
Depression domain													
• Baseline	7.20 (5.40)	6.09 (5.07)	6.02 (4.53)	0.495	‒	‒	0.907	‒	‒	1.000	‒	‒	0.442
• Change at week 4	−1.60 (5.34)	−1.35 (4.19)	−1.62 (4.80)	0.959	−0.25	−0.047	1.000	0.27	0.064	1.000	0.02	0.004	1.000
• Change at week 8	−4.08 (5.07)	−2.74 (4.74)	−2.02 (5.61)	0.193	−1.33	−0.263	0.725	−0.72	−0.152	1.000	−2.05	−0.405	0.221
Fatigue domain													
• Baseline	8.60 (3.61)	9.14 (3.22)	9.29 (3.17)	0.621	‒	‒	1.000	‒	‒	1.000	‒	‒	1.000
• Change at week 4	−2.45 (3.31)	−1.81 (3.59)	−1.17 (4.61)	0.330	−0.64	−0.192	1.000	−0.65	−0.180	1.000	−1.28	−0.387	0.411
• Change at week 8	−3.70 (3.79)	−3.98 (2.95)	−2.95 (3.36)	0.356	0.28	0.073	1.000	−1.02	−0.347	0.493	−0.75	−0.197	0.954
Confusion domain													
• Baseline	5.78 (4.01)	7.51 (3.77)	5.98 (3.17)	0.062	-	-	0.093	-	-	0.070	-	-	1.000
• Change at week 4	−1.05 (3.22)	−1.67 (4.27)	−1.02 (3.14)	0.642	0.62	0.194	1.000	−0.65	−0.152	1.000	−0.03	−0.008	1.000
• Change at week 8	−1.68 (3.58)	−2.70 (3.23)	−1.36 (3.45)	0.172	1.02	0.286	0.526	−1.34	−0.416	0.219	−0.32	−0.089	1.000
ERA domain													
• Baseline	12.55 (4.07)	12.00 (3.35)	12.57 (3.85)	0.731	‒	‒	1.000	‒	‒	1.000	‒	‒	1.000
• Change at week 4	−3.10 (4.37)	−1.33 (2.93)	−0.79 (3.76)	0.015	−1.77	−0.406	0.095	−0.54	−0.184	1.000	−2.31	−0.529	0.017
• Change at week 8	−3.40 (4.49)	−2.35 (4.48)	−0.88 (3.45)	0.025	−1.05	−0.234	0.759	−1.47	−0.328	0.321	−2.52	−0.561	0.021
Vigour domain													
• Baseline	8.10 (3.12)	7.56 (2.68)	8.93 (3.32)	0.118	‒	‒	1.000	‒	‒	0.143	‒	‒	0.884
• Change at week 4	0.65 (3.05)	0.67 (2.88)	0.55 (3.63)	0.982	−0.02	−0.008	1.000	0.13	0.044	1.000	0.10	0.034	1.000
• Change at week 8	2.50 (3.24)	1.19 (3.53)	1.05 (3.62)	0.118	1.31	0.405	0.262	0.14	0.039	1.000	1.45	0.448	0.182
POMS total score													
• Baseline	114.38 (18.33)	115.33 (20.74)	113.14 (18.68)	0.873	‒	‒	1.000	‒	‒	1.000	‒	‒	1.000
• Change at week 4	−6.20 (18.77)	−6.51 (15.56)	−5.98 (16.43)	0.989	0.31	0.017	1.000	−0.54	−0.034	1.000	−0.22	−0.012	1.000
• Change at week 8	−6.80 (18.09)	−6.09 (13.80)	−2.12 (19.67)	0.417	−0.71	−0.039	1.000	−3.97	−0.288	0.878	−4.68	−0.259	0.671

ARE, ashwagandha root extract; SHT, shatavari; PL, placebo; ERA, esteem-related affect; POMS, profile of mood states; SD., standard deviation.

* One-way analysis of variance (ANOVA) with Bonferroni *post hoc* pairwise comparisons.

** Between group *p*-value (ANOVA).

### 3.4 Perceived stress scale score, hot flashes, and mood improvement (*n* = 125)

**Figure 5:**
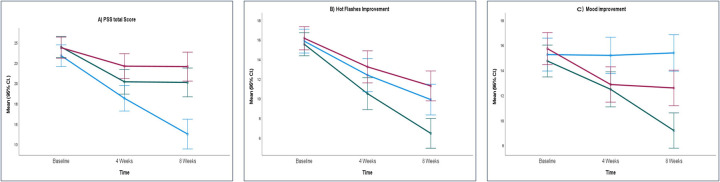
Mean scores of **(A)** PSS total score, **(B)** hot flashes improvement and **(C)** mood improvement. ARE, ashwagandha root extract; SHT, shatavari; PL, placebo; PSS, perceived stress scale; CI, confidence interval.

Table 4 shows that the total PSS score between the groups improved significantly in week 4 (*p* = 0.035) and week 8 (*p* < 0.0001). Hot flash frequency and severity decreased from baseline in all groups, with no significant between-group differences at week 4 (*p* = 0.171). By week 8, reductions were greater in the treatment groups, and a significant improvement was observed overall between groups (*p* = 0.002), with SHT showing a marked reduction compared to placebo (*p* = 0.002). There was a significant improvement seen in mood at week 8 (*p* = 0.008). The total change in PSS, hot flashes, and mood improvement for week 4 and week 8 are represented in Figure 5.

**Table 4:** PSS score, Hot flashes, and mood improvement at baseline and during the study period in the PP dataset (*n* = 125).

Parameters	ARE-SHT (*n* = 40)	SHT (*n* = 43)	PL (*n* = 42)	ANOVA	ARE-SHT vs. SHT			SHT vs. PL			ARE-SHT vs. PL		
	Mean (SD.)	Mean (SD.)	Mean (SD.)	“*p*”**	Mean Diff.	Effect size (d)	“*p*”*	Mean Diff.	Effect size (d)	“*p*”*	Mean Diff.	Effect size (d)	“*p*”*
PSS total score													
• Baseline	23.18 (6.70)	24.30 (3.73)	24.69 (4.27)	0.371	‒	‒	0.927	‒	‒	1.000	‒	‒	0.524
• Change at week 4	−5.33 (5.29)	−4.00 (4.49)	−2.45 (5.07)	0.035	−1.33	−0.250	0.143	−1.55	−0.344	0.337	−2.87	−0.543	0.002
• Change at week 8	−9.58 (8.42)	−4.12 (5.49)	−2.62 (4.05)	<0.0001	−5.46	−0.648	<0.0001	−1.50	−0.273	0.412	−6.96	−0.826	<0.0001
Hot flashes													
• Baseline	15.85 (3.96)	15.65 (3.11)	16.00 (4.83)	0.923	‒	‒	1.000	‒	‒	1.000	‒	‒	1.000
• Change at week 4	−3.55 (5.00)	−5.02 (4.83)	−2.86 (6.23)	0.171	1.47	0.295	0.648	−2.17	−0.448	0.200	−0.69	−0.139	1.000
• Change at week 8	−6.18 (5.71)	−9.07 (4.76)	−4.69 (6.55)	0.002	2.89	0.507	0.068	−4.38	−0.919	0.002	−1.48	−0.260	0.726
Mood improvement													
• Baseline	15.13 (3.35)	14.81 (3.71)	15.74 (5.13)	0.581	‒	‒	1.000	‒	‒	0.423	‒	‒	0.464
• Change at week 4	−0.28 (6.04)	−2.23 (4.85)	−2.69 (7.71)	0.189	1.96	0.324	0.480	0.46	0.094	1.000	−0.69	−0.139	0.256
• Change at week 8	−0.28 (6.84)	−5.42 (6.27)	−2.88 (8.91)	0.008	5.14	0.753	0.006	−2.54	−0.405	0.354	2.61	0.381	0.344

SD., standard deviation; ARE, ashwagandha root extract; SHT, shatavari; PSS, perceived stress scale.

* One-way analysis of variance (ANOVA) with Bonferroni *post hoc* pairwise comparisons.

** Between-group *p*-value (ANOVA).

Covariate-adjusted analyses indicated that FSH was a significant predictor for both PSS total score (*p* < 0.001) and mood improvement (*p* < 0.001), while group allocation also significantly influenced hot flash reduction (*p* = 0.006) and mood (*p* < 0.001). In contrast, age, BMI, and menopause status did not significantly affect outcomes. Adjusted mean scores with 95% confidence intervals are presented in Supplementary Table S3.

### 3.5 Serum hormonal levels and laboratory parameters (*n* = 125)

Tables 5, 6 summarize the serum hormonal levels and laboratory parameters. At week 8, ARE-SHT had higher estradiol levels [45.88 (13.03)] compared to PL [44.45 (14.83)]. Similarly, a numerical increase in FSH levels was observed in ARE-SHT [20.85 (10.71)] compared to PL [18.90 (9.39)]. LH levels remained stable throughout the study, while total testosterone showed a slight numerical increase by week 8; however, no significant between-group differences were observed. Also, no significant changes in the laboratory parameters were observed.

**Table 5:** Serum hormonal levels at baseline and week 8 in the PP dataset (*n* = 125).

Parameters	ARE-SHT (*n* = 40)	SHT (*n* = 43)	PL (*n* = 42)	ANOVA	ARE-SHT vs. SHT			SHT vs. PL			ARE-SHT vs. PL		
	Mean (SD.)	Mean (SD.)	Mean (SD.)	“*p*”**	Mean Diff.	Effect size (d)	“*p*”*	Mean Diff.	Effect size (d)	“*p*”*	Mean Diff.	Effect size (d)	“*p*”*
Estradiol (pg/ml)													
• Baseline	44.91 (13.37)	44.17 (13.61)	43.53 (15.09)	0.906	‒	‒	1.000	‒	‒	1.000	‒	‒	1.000
• Week 8	45.88 (13.03)	45.09 (13.29)	44.45 (14.83)	0.894	0.79	0.061	1.000	0.64	0.048	1.000	1.43	0.110	1.000
FSH (IU/L)													
• Baseline	19.88 (11.27)	18.66 (9.88)	17.98 (9.80)	0.701	‒	‒	1.000	‒	‒	1.000	‒	‒	1.000
• Week 8	20.85 (10.71)	19.58 (9.43)	18.90 (9.39)	0.660	1.27	0.119	1.000	0.68	0.072	1.000	1.95	0.182	1.000
LH (IU/L)													
• Baseline	13.60 (2.61)	13.88 (2.45)	13.76 (2.24)	0.871	‒	‒	1.000	‒	‒	1.000	‒	‒	1.000
• Week 8	14.09 (2.41)	14.34 (2.43)	14.22 (2.16)	0.888	−0.25	−0.104	1.000	0.12	0.049	1.000	−0.13	−0.054	1.000
Total Testosterone (ng/dl)													
• Baseline	21.13 (5.94)	22.25 (7.51)	19.86 (5.60)	0.232	‒	‒	1.000	‒	‒	0.265	‒	‒	1.000
• Week 8	22.76 (6.13)	23.10 (7.67)	21.74 (5.95)	0.619	−0.34	−0.055	1.000	1.36	0.177	1.000	1.02	0.166	1.000

SD, standard deviation; PP, per protocol; ARE/SHT, ashwagandha root extract/shatavari; SHT, shatavari; PL, placebo; FSH, follicle-stimulating hormone; IU/L, international units per liter; LH, luteinizing hormone; ng/dl, nanograms per deciliter; pg/ml, picograms per milliliter.

* One-way analysis of variance (ANOVA) with Bonferroni *post hoc* pairwise comparisons.

** Between group *p*-value (ANOVA).

**Table 6:** Laboratory parameters at baseline and week 8 in the safety dataset.

Parameters	ARE-SHT (*n* = 40)	SHT (*n* = 43)	PL (*n* = 42)	ANOVA	ARE-SHT vs. SHT			SHT vs. PL			ARE-SHT vs. PL		
	Mean (SD.)	Mean (SD.)	Mean (SD.)	“*p*”**	Mean Diff.	Effect size (d)	“*p*”*	Mean Diff.	Effect size (d)	“*p*”*	Mean Diff.	Effect size (d)	“*p*”*
Total Bilirubin (mg/dl)													
• Baseline	1.11 (0.10)	1.14 (0.07)	1.09 (0.08)	0.077	‒	‒	0.342	‒	‒	0.086	‒	‒	1.000
• Week 8	0.99 (0.32)	0.95 (0.36)	0.99 (0.40)	0.908	0.04	0.124	1.000	−0.03	−0.089	1.000	0.01	0.023	1.000
Direct Bilirubin (mg/dl)													
• Baseline	0.90 (0.11)	0.94 (0.06)	0.90 (0.08)	0.109	‒	‒	0.242	‒	‒	0.180	‒	‒	1.000
• Week 8	0.66 (0.21)	0.64 (0.24)	0.66 (0.27)	0.909	0.03	0.124	1.000	−0.02	−0.088	1.000	0.00	0.024	1.000
Indirect Bilirubin (mg/dl)													
• Baseline	0.20 (0.03)	0.20 (0.03)	0.19 (0.02)	0.286	‒	‒	1.000	‒	‒	0.864	‒	‒	0.367
• Week 8	0.33 (0.11)	0.32 (0.12)	0.33 (0.13)	0.923	0.01	0.115	1.000	−0.01	−0.080	1.000	0.00	0.024	1.000
ALP (IU/L)													
• Baseline	82.46 (23.93)	83.09 (27.52)	71.36 (18.95)	0.121	‒	‒	1.000	‒	‒	0.199	‒	‒	0.253
• Week 8	78.18 (20.98)	75.41 (16.38)	78.75 (15.34)	0.749	2.76	0.132	1.000	−3.34	−0.204	1.000	−0.57	−0.027	1.000
AST (IU/L)													
• Baseline	23.50 (4.61)	23.78 (4.78)	23.25 (5.54)	0.924	‒	‒	1.000	‒	‒	1.000	‒	‒	1.000
• Week 8	22.25 (3.86)	22.31 (5.72)	23.29 (3.72)	0.632	−0.06	−0.016	1.000	−0.98	−0.171	1.000	−1.04	−0.269	1.000
ALT (IU/L)													
• Baseline	18.62 (5.26)	18.47 (6.34)	25.45 (20.95)	0.074	‒	‒	1.000	‒	‒	0.134	‒	‒	0.154
• Week 8	18.86 (4.93)	20.79 (6.05)	19.00 (4.94)	0.319	−1.93	−0.392	0.530	1.79	0.295	0.632	−0.14	−0.029	1.000
Total Protein (g/dl)													
• Baseline	7.06 (1.04)	6.90 (1.11)	7.04 (0.75)	0.801	‒	‒	1.000	‒	‒	1.000	‒	‒	1.000
• Week 8	6.87 (0.81)	6.90 (0.80)	6.94 (0.67)	0.954	−0.03	−0.036	1.000	−0.03	−0.041	1.000	−0.06	−0.077	1.000
Albumin (g/dl)													
• Baseline	4.02 (1.03)	3.85 (0.81)	4.09 (0.75)	0.562	‒	‒	1.000	‒	‒	0.895	‒	‒	1.000
• Week 8	4.10 (0.85)	4.10 (0.85)	4.31 (0.78)	0.534	0.00	0.005	1.000	−0.22	−0.254	0.978	−0.21	−0.251	1.000
Globulin (g/dl)													
• Baseline	2.55 (0.57)	2.65 (0.67)	2.55 (0.58)	0.755	‒	‒	1.000	‒	‒	1.000	‒	‒	1.000
• Week 8	2.54 (0.78)	2.84 (0.69)	2.89 (0.62)	0.135	−0.30	−0.391	0.313	−0.04	−0.060	1.000	−0.34	−0.444	0.203
Creatinine (mg/dl)													
• Baseline	0.73 (0.20)	0.72 (0.17)	0.78 (0.19)	0.358	‒	‒	1.000	‒	‒	0.515	‒	‒	0.871
• Week 8	0.77 (0.17)	0.80 (0.12)	0.78 (0.15)	0.746	−0.03	−0.173	1.000	0.02	0.179	1.000	−0.01	−0.043	1.000
BUN (mg/dl)													
• Baseline	11.17 (3.63)	11.80 (4.27)	12.20 (4.82)	0.665	‒	‒	1.000	‒	‒	1.000	‒	‒	1.000
• Week 8	11.94 (3.15)	13.17 (3.52)	12.76 (3.34)	0.371	−1.23	−0.391	0.503	0.41	0.116	1.000	−0.82	−0.262	1.000
TSH (mIU/L)													
• Baseline	5.24 (2.26)	5.65 (2.40)	4.44 (2.40)	0.153	‒	‒	1.000	‒	‒	0.171	‒	‒	0.632
• Week 8	3.09 (1.43)	3.51 (1.02)	3.66 (1.02)	0.169	−0.43	−0.298	0.520	−0.15	−0.145	1.000	−0.57	−0.402	0.210
T3 (ng/dl)													
• Baseline	129.23 (26.23)	133.26 (25.27)	120.04 (14.54)	0.086	‒	‒	1.000	‒	‒	0.092	‒	‒	0.399
• Week 8	140.61 (32.09)	135.55 (26.75)	126.22 (17.53)	0.119	5.06	0.158	1.000	9.33	0.349	0.546	14.39	0.448	0.128
T4 (µg/dl)													
• Baseline	8.36 (2.25)	7.91 (2.56)	7.95 (1.94)	0.715	‒	‒	1.000	‒	‒	1.000	‒	‒	1.000
• Week 8	9.02 (1.71)	8.30 (2.04)	8.27 (2.10)	0.272	0.72	0.422	0.505	0.03	0.013	1.000	0.75	0.438	0.470

SD., standard deviation; PP, per protocol; ARE/SHT, ashwagandha root extract/shatavari; SHT, shatavari; PL, placebo; ALP, alkaline phosphatase; ALT, alanine transaminase; AST, aspartate transaminase; BUN, blood urea nitrogen; g/dl, grams per deciliter; IU/L, international units per liter; IU/ml, international units per milliliter; mIU/L, milli-international units per liter; mg/dl, milligrams per deciliter; ng/dl, nanograms per deciliter; pg/ml, picograms per milliliter; T3, triiodothyronine; T4, thyroxine; TSH, thyroid stimulating hormone; µg/dl, micrograms per deciliter.

* One-way analysis of variance (ANOVA) with Bonferroni *post hoc* pairwise comparisons.

** Between group *p*-value (ANOVA).

## 4 Discussion

Symptoms of perimenopause and post menopause can have a big impact on women's daily activities, productivity, and overall quality of life (20). Shatavari, a medicinal plant traditionally used in Ayurveda, contains phytoestrogenic compounds that have affinity for estradiol receptor-binding (21, 22). Along with the Shatavari root extract-alone arm, the Shatavari and Ashwagandha root extracts combination arm was included purely on an exploratory basis, with the intent to generate preliminary insights into potential additive effects. The study was not powered to draw definitive conclusions regarding the combination, and therefore, findings related to this arm should be considered hypothesis-generating. The primary objective of the study was to evaluate the efficacy of Shatavari root extract alone; however, the three-arm design also allowed for distinguishing the effects of Shatavari root extract alone and any additional benefits of the combination, thereby providing a more comprehensive clinical evaluation.

The study duration was set at 8 weeks, consistent with prior trials evaluating Ashwagandha and Shatavari in menopausal populations, which reported clinically meaningful improvements within a similar timeframe (12, 23). The dosing strategy was chosen to achieve a safe and balanced phytotherapeutic effect, avoiding excessive Ashwagandha exposure relative to Shatavari while ensuring clinical efficacy.

A study conducted by Gudise et al. (23), which was an eight-week, multicenter, interventional, prospective, randomized, double-blind, placebo-controlled trial on 70 females, reported a significant improvement in night sweats and hot flashes at follow-up visits on day 30 and day 60 (*p* < 0.0001). Though in the current study, hot flashes did not show significant improvement in week 4 but shown significance at week 8 between the groups. Despite this, it was observed that the somato-vegetative domain showed significant improvements in week 8 (*p* < 0.0001), as well as the psychosocial domain showed significant improvement in the SHT vs. PL (*p* < 0.0001) and ARE-SHT vs. PL (*p* < 0.0001) comparison. No significant change was observed between the ARE-SHT vs. SHT groups, suggesting that the combination is as effective as the SHT monotherapy. Gudise et al. (23), specifically focused on Shatavari monotherapy (500 mg/day), the current trial explored both Shatavari alone (300 mg/day) and a combination with Ashwagandha, thereby broadening the scope to include synergistic effects on mood and stress outcomes in addition to vasomotor symptoms.

A 12-week study conducted on 117 healthy women (aged 40–65 years), investigating a combination of *Tinospora cordifolia* (75 mg), *Asparagus racemosus* (100 mg), *Withania somnifera* (100 mg), and *Commiphora mukul* (225 mg) compared to a placebo (maltodextrin) was reported by Steels et al. (24). The results reported were significant improvements in the vasomotor (*p* < 0.001), psychosocial (*p* < 0.001), physical (*p* = 0.02), and sexual domains (*p* < 0.001) and in the overall MENQOL score. In contrast, the current study showed no significant improvements in the physical (*p* = 0.113), sexual domains (*p* = 0.238), or MENQOL total score (*p* = 0.117) between groups at week 8.

A 30-day trial by Leonard et al. (25) assessing the effect of ARE (225 mg/day) on cognitive function and mood reported, improvement in 60 participants demonstrating a significant difference in various POMS subdomains (*p* < 0.0001). In the current study, a significant baseline difference was observed in the POMS Confusion subscale between groups. To account for this imbalance, outcome analyses were adjusted for baseline Confusion scores along with other covariates (age, menopausal status, BMI, and FSH) using a generalized linear model. The intervention-related improvements in mood, stress (PSS), and emotional role functioning (ERA) remained consistent after adjustment, suggesting that the baseline difference in Confusion did not bias the overall results.

In the present study, ERA showed significant improvement at week 4 (*p* = 0.015) and week 8 (*p* = 0.025). Additionally, ARE-SHT vs. PL showed greater improvements compared to both SHT vs. PL and ARE-SHT vs. SHT, signifying an additive effect of ARE-SHT, which was comparable to or more effective than SHT alone.

A study conducted by Gopal et al. (12), on 100 women with climacteric symptoms demonstrated that ARE (300 mg BID) significantly reduced MENQOL scores (*p* < 0.0001), along with improvements in the somato-vegetative (*p* = 0.0152), psychological (*p* = 0.0003), and urogenital (*p* < 0.0001) domains. Gopal et al. (12) exclusively examined Ashwagandha at higher doses in perimenopausal women; the current trial tested a lower dose of Ashwagandha root extract (250 mg/day) in combination with Shatavari root extract and included a wider menopausal spectrum. This design allowed us to investigate not only single-agent but also combination effects, thereby providing novel insights into complementary phytotherapeutic approaches. The current study aligns with the study findings, a significant difference in PSS scores was observed in week 4 (*p* = 0.035) and week 8 (*p* < 0.0001). *Post hoc* analysis revealed a significant difference between ARE-SHT and SHT groups, suggesting combination therapy is more effective than monotherapy (12).

Based on the findings from both the previous and present studies indicated that ARE and SHT can work together to reduce the symptoms of stress, whereas the menopausal symptoms can be improved with either the combination or SHT alone. Mild adverse events were reported, including loose stools and dizziness in the ARE-SHT group, nausea in the SHT group, and headache in the PL group. However, these adverse events were mild, and the combination therapy was found to be safe and well (26, 27).

At week 8, participants in the ARE-SHT group demonstrated slightly higher estradiol levels and a modest numerical increase in FSH compared with placebo, although these changes were not statistically significant. LH levels remained stable, and total testosterone showed only a minor numerical increase. The absence of significant between-group differences suggests that the intervention did not meaningfully alter the hypothalamic-pituitary-gonadal (HPG) axis. From a clinical perspective, this is reassuring, as it indicates that the mood and quality-of-life benefits observed with SHT and ARE-SHT were not driven by major hormonal shifts, but rather through alternative mechanisms (e.g., adaptogenic, stress-modulating, or neuroendocrine pathways). Importantly, the stability of standard laboratory parameters further supports the safety of the intervention (11).

This study is robust as it employs a comparative analysis, allowing for a clear understanding of both the additive and individual effects of ARE and SHT. The observed additive effects of the ARE and SHT combination may be explained by the complementary pharmacological activities of both. ARE is well established as an adaptogen with the ability to modulate the HPA axis, balance cortisol levels, and improve stress resilience. It also exhibits neuroprotective and anti-inflammatory effects, which are related to improved mood and overall well-being. Whereas SHT contains steroidal saponins with phytoestrogenic activity, along with potent antioxidant and immunomodulatory properties, supporting hormonal balance and alleviation of vasomotor and urogenital symptoms in menopause. When administered together, these agents may act on both neuroendocrine and estrogenic pathways, thereby providing broader and more effective relief of menopausal symptoms than either intervention alone.

The three-arm study design is a cost-effective and time-efficient approach for evaluating multiple conditions simultaneously. There were no significant changes in safety parameters, and participants reported only mild adverse events, indicating that both the combination of ARE-SHT and SHT alone are safe and well-tolerated.

However, some limitations must be considered. Self-reporting bias may have influenced participants' reporting of adverse events, as they may have underreported or overreported symptoms based on personal perceptions. The study duration of 8 weeks, while adequate for assessing short-term efficacy and safety, limits conclusions regarding the sustained benefits and long-term safety of the intervention. Although the study was sufficiently powered for the primary outcomes, the sample size was relatively small and participants were recruited from specific geographic regions, which may limit the generalizability of the findings to broader and more diverse populations. The small sample size limited the feasibility of subgroup analyses by menopausal stage. Potential confounding influences from participants' diet, lifestyle, or physical activity were not systematically controlled, and although only mild adverse events were observed. In addition, as no adjustment for multiple testing was applied to exploratory hormonal analyses, there remains a potential risk of type I error, and these findings should be considered hypothesis-generating. Although FSH levels were measured, they were not systematically used to confirm the menopausal stage, which may limit the precision of participant characterization. Future studies with larger sample sizes, longer follow-up periods, and specific menopausal stages are warranted to evaluate the durability of effects and to identify any delayed adverse events.

## 5 Conclusion

The Shatavari root extract demonstrated significant reductions in MRS and PSS scores. While improvements in MENQOL scores, hot flashes, and hormonal changes were observed as trends, these did not reach statistical significance and therefore warrant confirmation in larger, longer-term studies. No changes in hepatic and renal markers were reported. There were no significant adverse events reported, indicating it is a safe and effective herbal option. However, the combination with Ashwagandha also showed improvement in most of the parameters as compared to Shatavari alone. Thus, Shatavari was found to be more effective alone and in combination with Ashwagandha in managing menopausal symptoms and in enhancing overall health, well-being, and quality of life in women with menopause. Future well-designed trials with larger and more diverse populations, extended follow-up durations, and incorporation of mechanistic biomarker assessments are recommended to confirm these findings and better understand the long-term efficacy and safety of Shatavari and its combinations.

## Data Availability

The raw data supporting the conclusions of this article will be made available by the authors, without undue reservation.
