# Supplementary material for: Efficacy and safety of Shatavari root extract (Asparagus racemosus) for menopausal symptoms: a randomized, double-blind, three-arm, placebo-controlled study
Source: Front Reprod Health. 2025 Nov 27;7:1654503. doi: 10.3389/frph.2025.1654503 (PMC12695842; doi:10.3389/frph.2025.1654503)
Supplement: Supplementary file 3 [file Table2.docx]

**Supplementary Table 2: Estimated POMS scores across time points (n=125)**

| **Parameters** | **Time**  **points** | **Unadjusted scores** | | **Adjusted**  **Score** | | |  |
| --- | --- | --- | --- | --- | --- | --- | --- |
| **Groups** |  | **Mean** | **SD.** | **Mean** | **95% CI.** | |  |
|  |  |  |  |  | *Lower* | *Upper* | *‘p’* |
| **Tension** |  |  |  |  |  |  |  |
| ARE+SHT | Baseline | 6.33 | 4.80 | 6.02 | 4.56 | 7.48 | 0.874 |
|  | Week 4 | 5.18 | 4.66 | 5.02 | 3.65 | 6.40 |  |
|  | Week 8 | 3.85 | 4.08 | 3.66 | 2.41 | 4.92 |  |
| SHT | Baseline | 6.40 | 5.39 | 6.50 | 5.08 | 7.92 |  |
|  | Week 4 | 5.12 | 4.33 | 5.18 | 3.85 | 6.51 |  |
|  | Week 8 | 4.00 | 4.47 | 4.18 | 2.97 | 5.39 |  |
| Placebo | Baseline | 6.21 | 4.64 | 6.40 | 4.97 | 7.82 |  |
|  | Week 4 | 5.05 | 4.11 | 5.13 | 3.79 | 6.46 |  |
|  | Week 8 | 4.19 | 3.45 | 4.19 | 2.96 | 5.40 |  |
| **Anger** |  |  |  |  |  |  |  |
| ARE+SHT | Baseline | 7.13 | 4.18 | 6.98 | 5.75 | 8.22 | 0.044 |
|  | Week 4 | 4.73 | 4.95 | 4.77 | 3.50 | 6.03 |  |
|  | Week 8 | 3.40 | 3.43 | 3.35 | 2.25 | 4.44 |  |
| SHT | Baseline | 5.74 | 3.17 | 5.81 | 4.62 | 7.00 |  |
|  | Week 4 | 4.70 | 3.03 | 4.61 | 3.38 | 5.84 |  |
|  | Week 8 | 3.40 | 2.86 | 3.46 | 2.40 | 4.52 |  |
| Placebo | Baseline | 7.14 | 4.37 | 7.21 | 6.01 | 8.41 |  |
|  | Week 4 | 5.90 | 3.77 | 5.95 | 4.72 | 7.19 |  |
|  | Week 8 | 5.29 | 3.97 | 5.27 | 4.21 | 6.34 |  |
| **Depression** |  |  |  |  |  |  |  |
| ARE+SHT | Baseline | 7.20 | 5.40 | 7.01 | 5.47 | 8.56 | 0.785 |
|  | Week 4 | 5.60 | 5.23 | 5.55 | 4.19 | 6.91 |  |
|  | Week 8 | 3.13 | 3.33 | 3.05 | 1.98 | 4.12 |  |
| SHT | Baseline | 6.09 | 5.07 | 6.11 | 4.61 | 7.61 |  |
|  | Week 4 | 4.74 | 3.92 | 4.63 | 3.31 | 5.95 |  |
|  | Week 8 | 3.35 | 3.74 | 3.37 | 2.34 | 4.41 |  |
| Placebo | Baseline | 6.02 | 4.53 | 6.19 | 4.68 | 7.69 |  |
|  | Week 4 | 4.40 | 3.97 | 4.57 | 3.24 | 5.89 |  |
|  | Week 8 | 4.00 | 3.08 | 4.05 | 3.01 | 5.09 |  |
| **Fatigue** |  |  |  |  |  |  |  |
| ARE+SHT | Baseline | 8.60 | 3.61 | 8.55 | 7.50 | 9.60 | 0.054 |
|  | Week 4 | 6.15 | 3.19 | 6.21 | 5.10 | 7.33 |  |
|  | Week 8 | 4.90 | 2.48 | 4.92 | 4.03 | 5.80 |  |
| SHT | Baseline | 9.14 | 3.22 | 9.18 | 8.17 | 10.19 |  |
|  | Week 4 | 7.33 | 3.05 | 7.33 | 6.25 | 8.40 |  |
|  | Week 8 | 5.16 | 2.66 | 5.18 | 4.32 | 6.04 |  |
| Placebo | Baseline | 9.29 | 3.17 | 9.29 | 8.27 | 10.31 |  |
|  | Week 4 | 8.12 | 4.25 | 8.06 | 6.98 | 9.15 |  |
|  | Week 8 | 6.33 | 3.18 | 6.30 | 5.44 | 7.16 |  |
| **Confusion** |  |  |  |  |  |  |  |
| ARE+SHT | Baseline | 5.78 | 4.01 | 5.63 | 4.48 | 6.77 | 0.048 |
|  | Week 4 | 4.73 | 2.90 | 4.69 | 3.74 | 5.64 |  |
|  | Week 8 | 4.10 | 2.80 | 4.05 | 3.20 | 4.90 |  |
| SHT | Baseline | 7.51 | 3.77 | 7.60 | 6.49 | 8.70 |  |
|  | Week 4 | 5.84 | 3.08 | 5.88 | 4.95 | 6.80 |  |
|  | Week 8 | 4.81 | 2.20 | 4.85 | 4.03 | 5.67 |  |
| Placebo | Baseline | 5.98 | 3.17 | 6.03 | 4.92 | 7.14 |  |
|  | Week 4 | 4.95 | 2.97 | 4.95 | 4.02 | 5.87 |  |
|  | Week 8 | 4.62 | 3.00 | 4.63 | 3.80 | 5.46 |  |
| **ERA** |  |  |  |  |  |  |  |
| ARE+SHT | Baseline | 12.55 | 4.07 | 12.77 | 11.72 | 13.82 | 0.022 |
|  | Week 4 | 9.45 | 3.29 | 9.57 | 8.51 | 10.63 |  |
|  | Week 8 | 9.15 | 2.87 | 9.22 | 8.19 | 10.25 |  |
| SHT | Baseline | 12.00 | 3.35 | 11.91 | 10.89 | 12.92 |  |
|  | Week 4 | 10.67 | 3.40 | 10.73 | 9.70 | 11.75 |  |
|  | Week 8 | 9.65 | 3.48 | 9.60 | 8.61 | 10.60 |  |
| Placebo | Baseline | 12.57 | 3.85 | 12.46 | 11.43 | 13.48 |  |
|  | Week 4 | 11.79 | 4.21 | 11.61 | 10.58 | 12.65 |  |
|  | Week 8 | 11.69 | 3.32 | 11.67 | 10.67 | 12.67 |  |
| **Vigour** |  |  |  |  |  |  |  |
| ARE+SHT | Baseline | 8.10 | 3.12 | 8.14 | 7.17 | 9.10 | 0.028 |
|  | Week 4 | 8.75 | 2.19 | 8.82 | 7.99 | 9.64 |  |
|  | Week 8 | 10.60 | 3.49 | 10.63 | 9.51 | 11.76 |  |
| SHT | Baseline | 7.56 | 2.68 | 7.50 | 6.57 | 8.43 |  |
|  | Week 4 | 8.23 | 2.71 | 8.22 | 7.42 | 9.02 |  |
|  | Week 8 | 8.74 | 3.41 | 8.70 | 7.61 | 9.79 |  |
| Placebo | Baseline | 8.93 | 3.32 | 8.96 | 8.02 | 9.89 |  |
|  | Week 4 | 9.48 | 2.94 | 9.42 | 8.62 | 10.23 |  |
|  | Week 8 | 9.98 | 3.82 | 9.99 | 8.89 | 11.09 |  |
| **POMS total score** | |  |  |  |  |  |  |
| ARE+SHT | Baseline | 114.38 | 18.33 | 113.29 | 107.47 | 119.11 | 0.732 |
|  | Week 4 | 108.18 | 20.04 | 107.85 | 102.53 | 113.18 |  |
|  | Week 8 | 107.58 | 18.30 | 106.17 | 101.28 | 111.05 |  |
| SHT | Baseline | 115.33 | 20.73 | 115.79 | 110.15 | 121.42 |  |
|  | Week 4 | 108.81 | 16.45 | 108.67 | 103.52 | 113.83 |  |
|  | Week 8 | 109.23 | 19.74 | 109.81 | 105.08 | 114.54 |  |
| Placebo | Baseline | 113.14 | 18.68 | 113.70 | 108.04 | 119.37 |  |
|  | Week 4 | 107.17 | 13.84 | 107.62 | 102.44 | 112.80 |  |
|  | Week 8 | 111.02 | 16.85 | 111.78 | 107.02 | 116.53 |  |
|  |  |  |  |  |  |  |  |

***Adjusted for covariates:*** *Tension: Age (p: 0.290); Menopause status (p: 0.895); BMI_V1 (p: 0.119); FSH_V1 (p: 0.002); Anger: Age (p: 0.951); Menopause status (p: 0.365); BMI_V1 (p: 0.432); FSH_V1 (p: 0.370); Depression: Age (p: 0.470); Menopause status (p: 0.164); BMI_V1 (p: 0.013); FSH_V1 (p: 0.095); Fatigue: Age (p: 0.735); Menopause status (p: 0.520); BMI_V1 (p: 0.524); FSH_V1 (p: 0.103); Confusion: Age (p: 0.464); Menopause status (p: 0.579); BMI_V1 (p: 0.207); FSH_V1 (p: 0.342); ERA: Age (p: 0.857); Menopause status (p: 0.338); BMI_V1 (p: 0.613); FSH_V1 (p: 0.000); Vigour: Age (p: 0.485); Menopause status (p: 0.170); BMI_V1 (p: 0.512); FSH_V1 (p: 0.061); POMS total score: Age (p: 0.630); Menopause status (p: 0.999); BMI_V1 (p: 0.098); FSH_V1 (p: <0.0001); ARE: Ashwagandha Root Extract: SHT: Shatavari; PSS: Perceived Stress Scale; MRS: Menopause Rating Scale; C.I: Confidence interval.*
