# Supplementary material for: Efficacy and safety of Shatavari root extract (Asparagus racemosus) for menopausal symptoms: a randomized, double-blind, three-arm, placebo-controlled study
Source: Front Reprod Health. 2025 Nov 27;7:1654503. doi: 10.3389/frph.2025.1654503 (PMC12695842; doi:10.3389/frph.2025.1654503)
Supplement: Supplementary file 4 [file Table3.docx]

**Table 3: Estimated PSS, Hot Flashes and Mood improvement across time points (n=125)**

| **Parameters** | **Time points** | **Unadjusted scores** | | **Adjusted**  **Score** | | | |  | |
| --- | --- | --- | --- | --- | --- | --- | --- | --- | --- |
| **Groups** |  | **Mean** | **SD.** | **Mean** | **95% CI.** | | |  | |
|  |  |  |  |  | *Lower* | *Upper* | | *‘p’* | |
| **PSS total Score** | |  |  |  |  |  |  | |  |
| ARE+SHT | Baseline | 23.18 | 6.70 | 23.38 | 22.04 | 24.71 | <0.0001 | |  |
|  | Week 4 | 13.60 | 5.92 | 18.15 | 16.58 | 19.71 |  | |  |
|  | Week 8 | 17.85 | 4.35 | 13.74 | 11.91 | 15.57 |  | |  |
| SHT | Baseline | 24.30 | 3.73 | 24.44 | 23.15 | 25.73 |  | |  |
|  | Week 4 | 20.19 | 6.02 | 20.17 | 18.65 | 21.69 |  | |  |
|  | Week 8 | 20.30 | 5.20 | 20.09 | 18.32 | 21.86 |  | |  |
| Placebo | Baseline | 24.69 | 4.27 | 24.36 | 23.06 | 25.66 |  | |  |
|  | Week 4 | 22.07 | 5.49 | 22.09 | 20.57 | 23.62 |  | |  |
|  | Week 8 | 22.24 | 6.85 | 22.03 | 20.25 | 23.81 |  | |  |
| ARE+SHT | Baseline | 15.85 | 3.96 | 15.84 | 14.63 | 17.06 | | 0.006 | |
|  | Week 4 | 12.30 | 4.98 | 12.38 | 10.69 | 14.06 | |  | |
|  | Week 8 | 9.68 | 5.29 | 9.88 | 8.31 | 11.44 | |  | |
| SHT | Baseline | 15.65 | 3.11 | 15.53 | 14.35 | 16.70 | |  | |
|  | Week 4 | 10.63 | 5.03 | 10.49 | 8.86 | 12.11 | |  | |
|  | Week 8 | 6.58 | 3.93 | 6.43 | 4.91 | 7.95 | |  | |
| Placebo | Baseline | 16.00 | 4.83 | 16.14 | 14.95 | 17.32 | |  | |
|  | Week 4 | 13.14 | 5.83 | 13.22 | 11.58 | 14.85 | |  | |
|  | Week 8 | 11.31 | 5.64 | 11.27 | 9.75 | 12.80 | |  | |
| **Mood improvement** | |  |  |  |  |  | |  | |
| ARE+SHT | Baseline | 15.13 | 3.35 | 15.24 | 13.93 | 16.55 | | <0.0001 | |
|  | Week 4 | 14.85 | 5.45 | 15.18 | 13.73 | 16.62 | |  | |
|  | Week 8 | 14.85 | 6.66 | 15.37 | 13.92 | 16.83 | |  | |
| SHT | Baseline | 14.81 | 3.71 | 14.73 | 13.46 | 16.00 | |  | |
|  | Week 4 | 12.58 | 4.97 | 12.47 | 11.07 | 13.87 | |  | |
|  | Week 8 | 9.40 | 5.31 | 9.18 | 7.77 | 10.59 | |  | |
| Placebo | Baseline | 15.74 | 5.13 | 15.72 | 14.44 | 16.99 | |  | |
|  | Week 4 | 13.05 | 5.45 | 12.85 | 11.44 | 14.26 | |  | |
|  | Week 8 | 12.86 | 6.80 | 12.58 | 11.16 | 13.99 | |  | |
|  |  |  |  |  |  |  | |  | |

***Adjusted for covariates:*** *Hot Flashes: Age (p: 0.129); Menopause status (p: 0.497); BMI_V1 (p: 0.240); FSH_V1 (p: 0.332); Group (p: 0.006); Mood improvement: Age (p: 0.174); Menopause status (p: 0.772); BMI_V1 (p: 0.718); FSH_V1 (p: <0.001); Group (p: <0.001). PSS total score:* *Age (p: 0.991); Menopause status (p: 0.950); BMI_V1 (p: 0.960); FSH_V1 (p: <0.001); ARE: Ashwagandha Root Extract: SHT: Shatavari; PSS: Perceived Stress Scale; MRS: Menopause Rating Scale; C.I: Confidence interval.*
