# Supplementary figures and images for: Efficacy and safety of Shatavari root extract (Asparagus racemosus) for menopausal symptoms: a randomized, double-blind, three-arm, placebo-controlled study
Source: Front Reprod Health. 2025 Nov 27;7:1654503. doi: 10.3389/frph.2025.1654503 (PMC12695842; doi:10.3389/frph.2025.1654503)

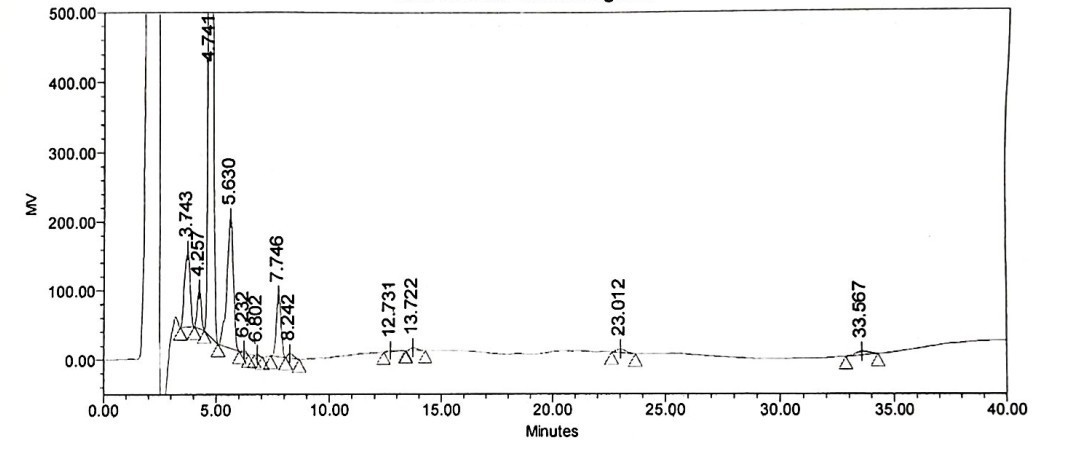

Supplement: Supplementary file 5 [file Image1.jpeg]

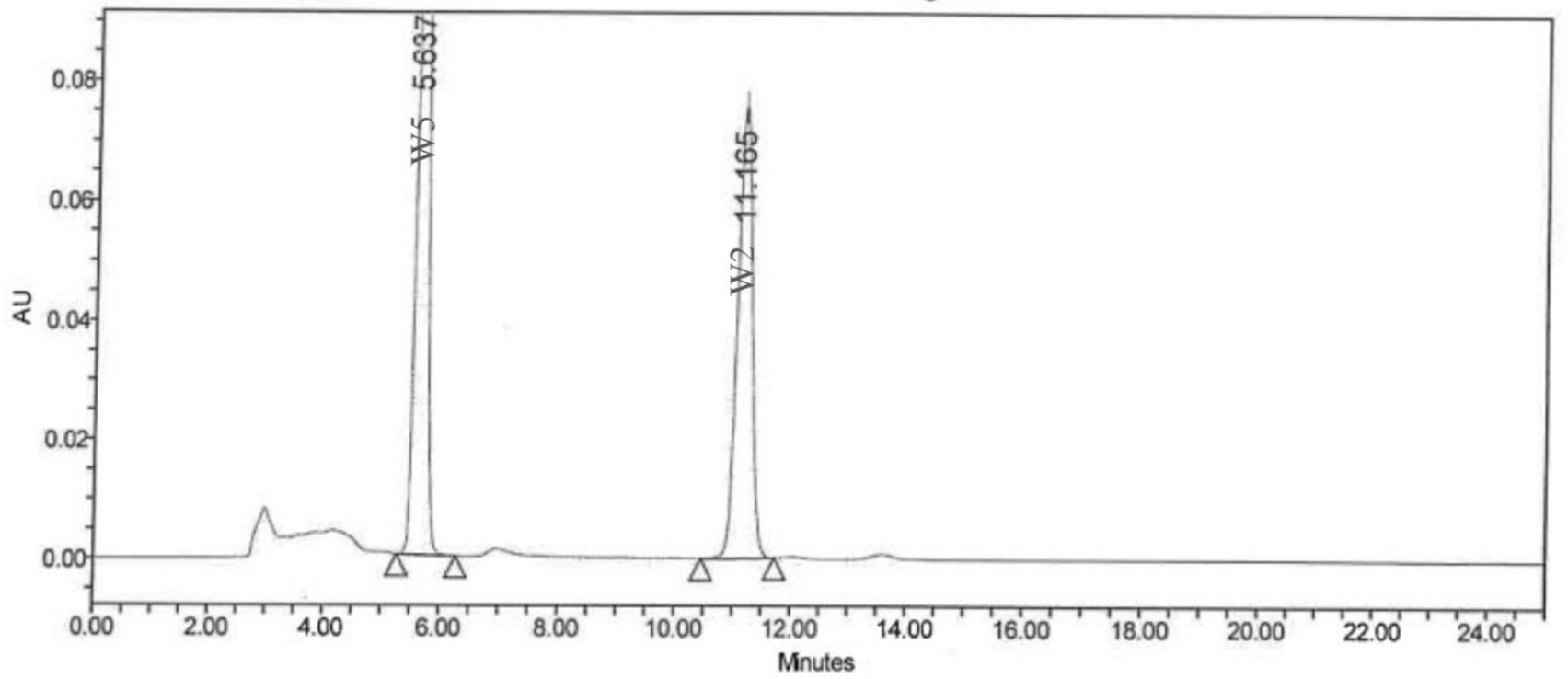

Supplement: Supplementary file 6 [file Image2.jpeg]
